# Efficacy of continuous local cryotherapy following total hip arthroplasty

**DOI:** 10.1051/sicotj/2019010

**Published:** 2019-05-03

**Authors:** Kentaro Iwakiri, Akio Kobayashi, Yuichi Takeuchi, Yusuke Kimura, Yoichi Ohta, Hiroaki Nakamura

**Affiliations:** 1 Department of Orthopaedic Surgery, Shiraniwa Hospital 6-10-1 Shiraniwadai Ikoma-city 630-0136 Nara Japan; 2 Department of Orthopaedic Surgery, Osaka City University Graduate School of Medicine 1-4-3 Asahi-machi Abeno-ku Osaka-city 545-8585 Osaka Japan

**Keywords:** Cryotherapy, Total hip arthroplasty, Swelling, VAS score, Patient satisfaction

## Abstract

*Background*: Cryotherapy is rarely reported on the usefulness of cryotherapy applied after total hip arthroplasty (THA), and there are no reports about patient satisfaction against the cryotherapy following THA. The aim of this study was whether cryotherapy can be useful for relieving pain, reducing blood loss, and swelling, and improving patient satisfaction after THA.

*Methods*: Thirty patients who had undergone THA were treated by a controlled cooling device for 72 h following THA (defined as the cryotherapy group). The other 30 patients without cryotherapy were not treated with cryotherapy (defined as the control group). Blood samples (creatine kinase, and C-reactive protein), estimated blood loss, visual analog scale (VAS) of pain score, total doses of diclofenac sodium suppository used for pain relief, thigh swelling, Western Ontario and McMaster Universities Osteoarthritis Index (WOMAC) score, and adverse outcomes were compared between two groups.

*Results*: Thigh circumference, measured on only day 4 postoperatively, was significantly lower in the cryotherapy than in the control group. Furthermore, patient satisfaction on postoperative days 4 and 7 was significantly higher in the cryotherapy than in the control group. There were no significant differences in other outcomes between groups.

*Conclusions*: These results support the potential benefit of cryotherapy for the reduction of swelling, and patient satisfaction during postoperative recovery of patients undergoing THA, even in the presence of periarticular injection and tranexamic acid administration for the prevention of postoperative pain and bleeding. Postoperative cryotherapy is a potentially simple, noninvasive, and relatively inexpensive option for post-THA management.

## Introduction

Total hip arthroplasty (THA) has become one of the mainstays for surgical management of end-stage arthritis of the hip [1]. In THA, cryotherapy has occasionally been reported to be useful for pain relief [2–5]. Regarding the mechanism underlying the effects of cryotherapy, the soft tissue is cooled to lower the intra-articular temperature, thereby reducing both the induction of neural signals and local blood flow [6]. Consequently, inflammatory responses are prevented. Thus, cryotherapy is considered to be useful for reducing blood loss and relieving local pain and swelling [7]. Moreover, cryotherapy has the advantages of being both safe and inexpensive. However, the majority of reports on cryotherapy describe the postoperative course after total knee arthroplasty (TKA) [8–10], with few reports on the usefulness of cryotherapy applied after THA [2–5]. Our present report describes the first survey to focus on postoperative swelling and patient satisfaction after THA.

This study aimed to examine whether continuous local cryotherapy is useful for relieving pain, and reducing blood loss, swelling, and the duration of hospitalization, as well as for improving patient satisfaction and postoperative outcomes after THA. We hypothesized that cryotherapy would reduce postoperative pain and improve patient satisfaction, thereby leading to more rapid progress with ambulatory rehabilitation.

## Materials and methods

We conducted a single-center retrospective study. This study protocol was approved by the institutional review board. All procedures performed in studies involving human participants were in accordance with the ethical standards of the institutional and/or national research committee and with the 1964 Helsinki declaration and its later amendments or comparable ethical standards.

### Study population

We reviewed 67 patients who had undergone primary THA for osteoarthritis or osteonecrosis of the hip at our institution between November 2012 and February 2015. We excluded patients who had simultaneous or staged bilateral THA or for revision THA. The subjects were stratified, according to the duration of the surgery from March to October in 2013 with our study group consisting of 30 patients (following their approval for participation) who had received cryotherapy and from November 2012 to February 2013 and after November 2013 with the control group consisting of 30 patients who did not receive cryotherapy. Seven patients were excluded without the approval for participation of the study. Demographic data are listed in Table 1.

**Table 1:** Demographic data of patients in two groups.

	Control	Cryotherapy	*p* value
*n*	30	30	
Female/male	29/1	29/1	–
Age (years)	67.6 ± 8.9	68.1 + 9.6	0.824*
BMI (kg/m^2^)	23.9 ± 3.1	24.1± 3.1	0.787*
OA/ION	29/1	29/1	–
Blood loss during surgery(mL)	391.2 ± 173.5	451.1 ± 240.6	0.274*
Duration of surgery (min.)	96.6 ± 15.6	100.4 ± 16.7	0.367*
Hospital stay (day)	24.7 ± 7.0	25.7 ± 5.6	0.558*

Data are shown as mean ± standard deviation or numbers.

OA, osteoarthritis; ION, idiopathic osteonecrosis of the femoral head.

* Mann–Whitney *U* test.

### Surgery

All patients underwent cementless THA via an anterolateral approach in the lateral position under general anesthesia. All cases were performed by a single surgeon. All patients routinely administered an intraoperative periarticular injection that we have previously reported (40-mL cocktail including ropivacaine, epinephrine, ketoprofen, methylprednisolone sodium, and normal saline solution) [11]. As prevention for perioperative bleeding, 2 g of tranexamic acid was injected (1 g intravenously before surgery and 1 g under the tensor fascia tissue after suture of the tensor fascia tissue). None of the patients had a drainage catheter placed. Wound closure strips (LEUKOSTRIP, Smith and Nephew, USA) were applied to the suture wound and covered with a transparent dressing of absorbent foam (OPSITE Post-Op Visible, Smith and Nephew, USA).

### Interventions

**Figure 1:**
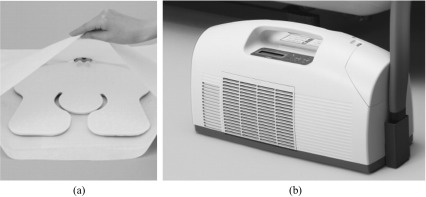
(a) A cooling pad (28 × 29 cm) wrapped in a waterproof cover for the surgical wound and the thigh (Sigmax, Tokyo, Japan). (b) A computer-controlled cooling device (Icing System CF3000, Sigmax, Tokyo, Japan).

Postoperatively in the cryotherapy group, a cooling pad (28 × 29 cm) wrapped in a waterproof cover was applied to the dressing immediately after surgery (Figure 1a), and the surgical wound and the thigh were fixed with a commercially available cloth anchor bandage (Sigmax, Tokyo, Japan). A controlled cooling device (Icing System CF3000, Sigmax, Tokyo, Japan) (Figure 1b) was initiated immediately after surgery and operated constantly for 72 h at the cooling temperature of 5 °C. The subjects were instructed to wear the cooling device whenever on bed. The same procedure was treated for the control group, involving the fixation with the cloth anchor bandage for 72 h without the cooling procedure. The intermittent compression device for thrombosis prophylaxis was used for 24 h immediately after surgery.

### Perioperative medications

For all subjects, a nonsteroidal anti-inflammatory drug (200 mg of celecoxib) was administered twice a day for the first 7 days after surgery. The diclofenac sodium suppository (25 mg) was utilized for the rescue analgesia. The cephalosporin was intravenously administered perioperatively as an antibiotic prophylaxis and every 6 h for 24 h after surgery. No thrombo-prophylaxis was treated. Every patient received an autologous blood transfusion (400 mL) on postoperative day 1.

### Outcome measurements

#### Primary outcomes

Primary outcomes included laboratory data (creatine kinase [CK], and C-reactive protein [CRP] obtained preoperatively and on postoperative days 1, 4, 7, 14, and 21), estimated blood loss [12], visual analog pain score on motion obtained once a day during physical therapy (VAS on motion obtained preoperatively and on postoperative days 4, 7, 14, and 28), and thigh swelling (thigh circumference measured at 5 cm proximal to the patella superior border obtained preoperatively and on postoperative days 4, 7, 14, and 28). The measurements of VAS score and thigh circumference were calculated by the physical therapist in the blinded fashion.

The estimated blood loss was calculated by the difference between the preoperative hemoglobin and the lowest postoperative level. The estimated blood loss was measured by the Nadler formula [12] at postoperative day 4 after surgery. The VAS score was defined from 0 mm (indicating no pain) to 100 mm (indicating extreme pain) in 10-mm increments. It was also recorded if a diclofenac sodium suppository was used as rescue analgesia within 21 days postoperatively. The ratio of postoperative thigh circumference to preoperative thigh circumference was measured to compare thigh swelling between the two groups.

#### Secondary outcomes

Secondary outcomes measured were the WOMAC Index (obtained preoperatively, and 3 months postoperatively), patient satisfaction of walking ability (VAS score obtained on postoperative days 4, 7, 14, and 28) and adverse events during this study, including surgical site infection, peroneal nerve palsy, wound complications, and deep venous thrombosis (DVT). The WOMAC Index score ranged from 100 points (indicating poor assessment) to 0 points (indicating excellent assessment for pain, stiffness, and function). The VAS score of patient satisfaction of walking ability ranged from 0 mm (indicating poor satisfaction) to 100 mm (indicating excellent satisfaction) in 10-mm increments [1].

### Statistical analysis

The Mann–Whitney *U*-test for continuous variables and Fisher’s exact probability test for categorical variables were used to analyze the primary and secondary outcomes statistically between the two groups. The *p* value of less than 0.05 was considered to indicate a statistically significant difference. All statistical analyses were done using the SPSS statistics version 22.0 (IBM Corp., USA).

## Results

Thirty patients in the cryotherapy group and thirty patients in the control group were included and compared in the study (Table 1). The average age of all subjects at surgery was 67.9 ± 9.2 years. There were 58 women and 2 men. The preoperative diagnosis was osteoarthritis of the hip in 58 patients and idiopathic osteonecrosis of the hip in two patients. There were no significant differences in age (*p* = 0.824), BMI (*p* = 0.787), blood loss during surgery (*p* = 0.274), the duration of surgery (*p* = 0.367), or duration of hospitalization (*p* = 0.558) between the two groups (Table 1).

### Primary outcomes

There were no significant differences between the two groups in CK levels, CRP levels, estimated blood loss, or pain VAS scores. However, the total amount of diclofenac sodium suppository tended to be significantly lower in the cryotherapy group (*p* = 0.070). Thigh circumference, measured postoperatively only on day 4, was significantly lower in the cryotherapy group than in the control group (*p* = 0.045) (Table 2). The overall VAS reduction was higher in the control group than in the cryotherapy group, but there were no significant differences between the two groups (*p* = 0.52).

**Table 2:** Primary outcomes.

	Control	Cryotherapy	*p* value
Laboratory data			
CK (IU/L)			
Pre-op.	96.6 ± 63.7	107.0 ± 79.4	0.578
Day 1	439.1 ± 201.4	467.1 ± 234.8	0.622
Day 4	447.1 ± 233.6	525.7 ± 266.8	0.230
Day 7	183.2 ± 106.8	181.9 ± 144.5	0.054
Day 14	53.3 ± 13.6	67.5 ± 38.9	0.067
Day 21	57.3 ± 15.4	60.5 ± 31.7	0.668
CRP (mg/dL)			
Pre-op.	0.21 ± 0.24	0.13 ± 0.15	0.145
Day 1	1.93 ± 0.93	1.50 ± 0.69	0.056
Day 4	4.59 ± 3.05	4.57 ± 2.74	0.971
Day 7	1.27 ± 1.06	1.42 ± 1.02	0.574
Day 14	0.38 ± 0.37	0.44 ± 0.68	0.701
Day 21	0.28 ± 0.27	0.32 ± 0.48	0.719
Estimated blood loss (mL)	784.7 ± 293.3	699.6 ± 296.2	0.268
Pain VAS			
Pre-op.	29.3 ± 33.9	24.1 ± 28.5	0.536
Day 4	9.3 ± 13.6	11.5 ± 19.3	0.624
Day 7	7.1 ± 9.7	11.5 ± 17.3	0.243
Day 14	5.4 ± 9.4	9.4 ± 17.8	0.294
Day 28	1.2 ± 2.2	3.3 ± 7.1	0.342
Thigh girth (the ratio of Post/Pre)			
Pre-op.	1	1	
Day 4	1.067 ± 0.055	1.041 ± 0.038	0.045
Day 7	1.047 ± 0.057	1.062 ± 0.045	0.267
Day 14	1.006 ± 0.030	1.021 ± 0.048	0.156
Day 28	1.007 ± 0.026	0.946 ± 0.270	0.246
Total amount of dicrofenac sodium suppository (mg)			
	72.2 ± 79.5	31.7 ± 60.9	0.070

Data are shown as mean ± standard deviation.

### Secondary outcomes

There were no significant differences in WOMAC scores between the two groups. However, patient satisfaction of walking ability measured on postoperative days 4 and 7 was significantly higher in the cryotherapy group than in the control group (*p* = 0.03 and 0.04, respectively). No complications such as skin problems, infection, symptomatic DVT, or neuroparalysis were observed in either group (Table 3).

**Table 3:** Secondary outcomes.

	Control	Cryotherapy	*p*-Value
WOMAC			
Pre-op	37.0 ± 17.0	38.9 ± 20.5	0.698
Post-op 3 months	15.0 ± 11.8	13.9 ± 10.6	0.408
Patient-satisfaction of walking ability			
Day 4	38 ± 25	62 ± 16	0.030
Day 7	48 ± 33	67 ± 17	0.040
Day 14	51 ± 31	67 ± 20	0.080
Day 28	61 ± 31	75 ± 20	0.120
Adverse events			
Wound complications	0	0	–
Surgical site infections	0	0	–
Nerve palsy	0	0	–
Deep venous thrombosis	0	0	–

Data are shown as mean ± standard deviation or numbers.

## Discussion

In recent years, cryotherapy after TKA, shoulder arthroscopic surgery, and carpal tunnel release has sporadically been reported to be useful for postoperative pain relief and prevention of swelling [8–10]. However, only a few reports have described the effects of continuous cryotherapy applied to the hip joints, which are located deeper than the knee and hand joints, following THA [2–5].

Saito et al. reported the use of continuous cryotherapy after THA to be effective for pain relief (according to VAS score) only in the early postoperative period, while no useful effects were noted for hematological data (i.e., blood loss, CK, and CRP) [4]. Moreover, Leegwater et al. reported that a combination of cryotherapy and compression slightly reduced bleeding in the early postoperative period [2]. In the present study, we, for the first time, have added thigh swelling and patient satisfaction of walking ability assessments in order to clarify the effects of cryotherapy. Contrary to previous reports, there were no significant differences in pain VAS scores and estimated blood loss between the control and cryotherapy groups. Otherwise, on postoperative day 4, swelling was significantly suppressed, based on thigh circumference, and the total amount of diclofenac sodium suppository given tended to be reduced in the cryotherapy group. Because a periarticular multimodal injection was used in all patients for postoperative pain relief at our institution, the VAS scores might have been kept low enough to mask any difference between the two groups.

The mechanisms by which cryotherapy relieves pain and prevents swelling are considered to involve the constriction of blood vessels to reduce blood flow and edema [6]. Moreover, cryotherapy prevents inflammation by lowering tissue metabolic rates and inhibiting enzymatic activity that was representative in prostaglandin E_2_ as an indicator of pain and inflammation [13]. Consequently, secondary tissue damage is prevented. The prevention of postoperative swelling of the lower limbs reduces the incidence of thrombogenesis in the early postoperative period [14]. At our institution, tranexamic acid was injected intravenously before surgery and beneath the tensor fascia tissue after suture of the tensor fascia tissue to all patients for the prevention of postoperative bleeding, and none of the patients had a drainage catheter placed for the improvement of patient satisfaction. In the present study, significant prevention of swelling, as evidenced by reduced thigh circumference on postoperative day 4, can presumably be attributed to the use of cryotherapy. As bleeding and swelling were mild in all patients because of the effect of the tranexamic acid injection and periarticular cocktail injection, the effects of cryotherapy might have been less evident [15, 16].

There were a few limitations in this study. First, the patients in the cryotherapy group were instructed to wear the cooling device whenever on bed, but there was variation in the duration of cooling time over the 72-hour period. This factor was not considered in this study. Secondly, this was a retrospective study. Third, the thigh circumference at 5 cm proximal to the patella superior border was measured. It is unknown how much the thigh circumference at 5 cm proximal to the patella superior border reflects thigh swelling. Fourth, there is no evidence whether the cooling temperature of 5 °C is suitable for the cryotherapy. Last, there might be potentially some bias in the improved satisfaction scores for patients who were treated with cryotherapy. Any other intervention except for cryotherapy might have been associated with improved satisfaction.

The present results revealed that continuous cryotherapy is useful for reducing swelling, and improving patient satisfaction of walking ability, even when a peri-articular multimodal cocktail is injected for postoperative pain relief or tranexamic acid is injected for the prevention of postoperative bleeding. Thus, cryotherapy may allow early postoperative rehabilitation and consequently reduce the risk of postoperative DVT.

Postoperative cryotherapy is a potentially simple, noninvasive, and relatively inexpensive option for post-THA management.

## Conclusion

This study examined whether continuous cryotherapy can be useful for relieving pain, and reducing blood loss, swelling, patient satisfaction, and the duration of hospitalization after THA. Continuous cryotherapy was effective in the immediate postoperative period for reducing swelling and minimizing the use of diclofenac sodium suppository, and improving patient satisfaction of walking ability, even when a periarticular multimodal cocktail was injected for postoperative pain relief or tranexamic acid was injected for prevention of postoperative bleeding.

## Conflict of interest

I certify that each of us has no financial conflict of interest (e.g., consultancies, stock ownership, equity interest, patent/licensing arrangements, etc) in connection with this article.
